# Supplementary material for: Improving Cuticle Thickness and Quality Traits in Table Grape cv. ‘Italia’ Using Pre-Harvest Treatments
Source: Plants (Basel). 2024 Aug 28;13(17):2400. doi: 10.3390/plants13172400 (PMC11396816; doi:10.3390/plants13172400)
Supplement: Supplementary file 1 [file plants-13-02400-s001.zip › plants-3156156-supplementary.pdf]

Texture Profile Analyses of 'Italia' berries

Table S1. TPA data (Texture Profile Analyses) of 'Italia' berries at the phenological stage BBCH89 (complete ripening). Data presented as means. Different letters indicate significant differences (*p*-value < 0.05). SA: salicylic acid, AN: A. nodosum, G: Girdled, U: Ungirdled.

Treatment	Hardness	Cohesiveness	Springiness	Chewiness
$CaCl_2$_G	20.727 a	0.202 a	1.867 a	7.694 a
$CaCl_2$_U	18.261 a	0.218 a	1.743 a	6.863 a
$CaCl_2$+AN_G	23.101 a	0.210 a	1.765 a	8.600 a
$CaCl_2$+AN_U	22.741 a	0.197 a	1.888 a	8.370 a
$CaCl_2$+SA_G	22.160 a	0.700 a	1.343 a	7.958 a
$CaCl_2$+SA_U	19.806 a	0.215 a	1.723 a	7.326 a
Untreated_G	21.227 a	0.191 a	1.937 a	7.724 a
Untreated_U	22.168 a	0.211 a	1.351 a	6.357 a

Total soluble solid and titratable acidity in % of Tartaric acid of 'Italia' berries

Table S2. TSS (total soluble solids) and TA (titratable acidity) of 'Italia' berries at the phenological stage BBCH89 (complete ripening). Data presented as means ± (SE). Different letters indicate significant differences (*p*-value < 0.05). SA: salicylic acid, AN A. nodosum, G: girdled, U: Ungirdled.

Treatment	TSS (°Brix)	TA (%)
$CaCl_2$_G	16.54±(0.20) a	0.86±(0.04) a
$CaCl_2$_U	16.84±(0.12) a	0.81±(0.02) a
$CaCl_2$+SA_G	16.69±(0.15) a	0.84±(0.02) a
$CaCl_2$+SA_U	16.80±(0.16) a	0.80±(0.02) a
$CaCl_2$+AN_G	17.03±(0.16) a	0.79±(0.01) a
$CaCl_2$+AN_U	17.10±(0.07) a	0.87±(0.06) a
Untreated_G	16.63±(0.13) a	0.79±(0.02) a
Untreated_U	16.57±(0.17) a	0.77±(0.02) a
